# Does the Scene Presentation of AI-Generated Photos Affect Travel Intention? An Empirical Study Drawing on Construal Level Theory

**DOI:** 10.3390/bs16071220

**Published:** 2026-07-18

**Authors:** Shanxin Wu, Yanda Huo, Xuedong Liang, Peng Luo

**Affiliations:** 1 Adam Smith Business School, University of Glasgow, Glasgow G11 6EY, UK; 3075125w@student.gla.ac.uk; 2 Business School, Sichuan University, Chengdu 610064, China; liangxuedong@scu.edu.cn

**Keywords:** AI-generated photo, scene presentation, construal level theory, destination image, occupationally generated content, user-generated content

## Abstract

The tourism industry is witnessing an increase in innovative AI-generated content (AIGC). AI-generated images are a key visual form of AIGC, and this study focuses on a specific subset of them: AI-generated images that realistically depict destination scenery, which we define as AI-generated photos (AIGP). Based on construal level theory, this study explored the visual cues of AIGP. Two empirical studies were conducted to examine the influence of scene presentation within AIGP on tourists’ travel intention. The findings revealed that panoramic series in AIGP, as opposed to close-up series, significantly enhanced travel intention. The results further suggested an indirect association through destination images and a moderating role of AIGP source. Specifically, the effects of scene presentation were found to be diminished when an AIGP was sourced from user-generated content. This study makes several notable contributions. First, it expands the application of AIGP within the tourism sector. Second, it enriches the literature on visual cues and destination images by delineating the process through which AIGP scene presentation influences travel intention. Third, the research clarifies the moderating role of the AIGP source, expanding the boundary of AIGP effects. These insights add depth to the dual fields of social media and destination marketing, highlighting the contextual variability in the efficacy of AIGC-driven marketing strategies.

## 1. Introduction

Leveraging the high efficiency and novel experiences provided by AI-generated content (AIGC), significant impacts are being made on the tourism industry (Wong et al., 2023). In tourism marketing, images play a crucial role. For instance, travel photos from tourists can inform and inspire others by conveying a firsthand destination experience, while photos generated by destination marketing organizations (DMOs) can quickly capture social media users’ attention (H. Kim et al., 2024; Yim et al., 2021). However, obtaining satisfactory photos often proves challenging due to the necessity of ideal scenes, suitable weather conditions, and advanced photography skills, often necessitating photo editing to compensate (N. Li et al., 2023). AI-generated images can address the limitations of real-life photography by pre-setting angles, backgrounds, and weather conditions, significantly reducing the time and cost associated with on-site shooting. Currently, on social media platforms such as X, Weibo, and TikTok, the low technical barrier and high operability of generative AI technologies have led to a surge in personal users posting AI-generated virtual landscapes, while some DMOs have also started experimenting with AIGC for promotional purposes. Chengdu Metro, for instance, has used generative AI technologies like Sora and Midjourney to promote concept metro stations on social media, garnering significant views^1^. We aim to provide a more intuitive and straightforward reference for the application of AI-generated images in the tourism industry that, similar to photographs, present a realistic visual representation of destination scenery. Following the research by Göring et al. (2023), we define these images as AI-generated photos (AIGP), which can be used to enhance tourists’ satisfaction and loyalty and reduce the costs for DMOs, greatly benefiting tourism businesses (Ku & Chen, 2024).

To avoid terminological ambiguity, we clarify the relationship among three related terms used in this paper: AI-generated content (AIGC) is the broadest category, encompassing all AI-generated outputs such as text, images, and video; AI-generated images form the visual subset of AIGC; and AI-generated photos (AIGP) are a specific type of AI-generated image that realistically represents destination scenery, similar to conventional photographs. Throughout this paper, AIGP refers specifically to the study stimulus, whereas AIGC and AI-generated image are used only when referring to these broader categories. It should be noted that realistic here refers to the photorealistic visual style of the images rather than to the depiction of any specific existing destination; the scenes themselves may be either real or fictional.

Since the release of ChatGPT 3.5 in 2022, the tourism industry has witnessed an increase in innovative AIGC. However, research on AI-generated images, and AIGP in particular, in the tourism field remains insufficient for three main reasons. Firstly, AI-generated images themselves have received relatively limited attention, as most existing studies focus more on the impact of text generated by large language models (LLMs) such as ChatGPT (Carvalho & Ivanov, 2024; Iskender, 2023). Secondly, vivid and intuitive images can have a positive impact on consumer attitudes, with visual cues in images consistently being a significant factor in consumer decision-making (Wang et al., 2024). However, current research on AI-generated images mostly focuses on visual feature recognition, with very few studies examining the specific impact of visual cues from AI-generated images on viewers (Göring et al., 2023; Z. Zhang et al., 2023). Thirdly, concerning specific visual cues within images, existing research primarily addresses the effects of image color, color temperature, and implied motion (Fink et al., 1996; H. Kim et al., 2024; F. S. Li & Ma, 2024). Only Su and Li (2024) have explored the role of spatial distance as a visual cue in the tourism context; however, currently, we have identified limited research extending this line of inquiry to AI-generated images. Indeed, due to technological and hardware limitations, the quality of AI-generated images varies significantly (Z. Zhang et al., 2023). Many AI-generated images, especially those not refined or filtered in post-production, still have detail defects that need improvement. The proximity or distance in these images may magnify or minimize certain defects, leading to different impacts. Thus, spatial distance as a visual cue in AI-generated images is a highly valuable yet under-researched topic.

The scene presentation serves as a representative spatial distance among visual cues in photography (Wang et al., 2024). To address the aforementioned research gaps, this study examines whether the scene presentation of AIGP influences tourists’ perceptions of destinations and their travel intention. Additionally, we incorporate the critical process variable of destination image (DI) in tourism marketing as a mediating variable along this influence path. This approach provides a more detailed illustration of tourists’ perceptions of destinations and the psychological journey leading to their travel intentions. AIGP are categorized into panoramic and close-up series. Based on construal level theory (CLT), we hypothesize that panoramic-series AIGP have a stronger positive impact on tourists’ DI and travel intention compared to close-up-series AIGP. Additionally, as social media encompasses various sources of content generation, it is essential to study both the role of AIGP in destination marketing and the impact of their sources. DMOs, from an official perspective, release videos, photographs, and website information, categorized as occupationally generated content (OGC). Conversely, more independent travelers use their personal accounts to share user-generated content (UGC). Research indicates that OGC and UGC differently influence tourists’ perceptions and intentions (Deng & Qu, 2022). However, there is a lack of studies on the distinctions between occupationally generated AIGP and user-generated AIGP. Based on this, the present study also investigates whether the impact of AIGP scene presentation is moderated by its source.

This study makes significant contributions. Firstly, it extends the application of AI-generated images in the tourism sector by empirically confirming the influence of scene presentation in AIGP on tourists’ intentions. Second, it contributes to the literature regarding visual cues and destination images by elucidating the process through which AIGP scene presentation, as a form of visual cue, facilitates DI, thereby positively influencing travel intention. Finally, this study elucidates the moderating role of the AIGP source, differentiating between OGC and UGC. It unveils the boundary conditions under which the effects of AIGP are operational, thereby making a substantial contribution to the intertwined domains of social media and destination marketing.

## 2. Theory Background

### 2.1. Construal Level Theory

Construal level refers to the degree of abstraction in goal-directed behavior within cognitive processes (Liberman & Trope, 1998). Psychological distance is considered to be a broad, multifaceted concept that explains individuals’ perceptions of objects as near or far across dimensions such as time, space, social relationships, and probability (Soderberg et al., 2015). CLT synthesizes these two concepts, with its core idea being that people’s subjective sense of psychological distance from an object influences their preferences, decisions, and behaviors (Trope et al., 2007). Specifically, CLT posits that the human brain tends to perceive and organize information based strictly on psychological distance (Trope & Liberman, 2010). When an individual perceives an object as distant, they operate at a high construal level, focusing on the abstract and central features of the object. Conversely, when the object is perceived as close, the individual operates at a low construal level, concentrating on the concrete and peripheral features of the object.

Currently, a substantial body of empirical research supports CLT, which is frequently employed in the realm of consumer behavior to elucidate consumption decisions. For instance, consumers operating at a high construal level exhibit a higher purchase intention when exposed to abstract advertising content, whereas those at a low construal level show a higher purchase intention when exposed to concrete advertising content (Dogan & Erdogan, 2020; Scarpi, 2021).

### 2.2. Scene Presentation

Scene presentation is a photography term that refers to the varying presentation of the subject’s scope within a photographic work by adjusting the shooting distance while maintaining a fixed focal length (Wang et al., 2024). It typically includes close-up, medium close-up, medium shot, long shot, and extreme long shot (Su & Li, 2024). According to the scene presentation classification theory, close-ups, medium close-ups, and medium shots are considered part of the close-up series, whereas long shots and extreme long shots belong to the panoramic series (Wang et al., 2024). Scene presentation is a crucial factor in eliciting emotional responses from viewers (Benini et al., 2010). Different scene presentations can convey various artistic effects in terms of spatial range and visual style: close-ups emphasize the inner world of characters, achieving the goal of conveying information and enhancing visual impact, while panoramic scenes present a broad scene, often used to amplify the work’s emotional appeal (Philbrick, 2004). Hence, scene presentation possesses both narrative and lyrical functions, requiring creators to select appropriate scene presentations based on their creative intentions during shooting or image creation.

Scene presentation traditionally pertains to actual photographs. In our study, however, we focus on AIGP, which are designed to present photorealistic visual representations of plausible destination scenery, regardless of whether the depicted destination is real or fictional. The concept of scene presentation for AIGP aligns with that for real photographs. Consequently, we have appropriately applied the concept of scene presentation to AIGP in our research. At the same time, defining a specific scene in detail is inherently challenging, and the uncertainties in the generation process of AIGP further exacerbate this difficulty. Therefore, following the paradigm of advertising marketing research, we categorize AIGP scenes into close-up series and panoramic series, rather than changing details within a specific scene (Wang et al., 2024).

### 2.3. Destination Image

The image of a tourist destination refers to the overall impression of the destination formed by the perception and evaluation of destination information by potential tourists (Qu et al., 2011). While playing an important role in research about tourist decision-making and subsequent behavior, DI has also been recognized by the academic community as a multidimensional concept that is the subject of multiple attention research in the social science literature (Jalilvand et al., 2012). Gartner (1994) first proposed that DI is a three-dimensional structure, while Baloglu and McCleary (1999) proposed a ‘new three-dimensional structure’ that includes cognitive destination image (CDI), affective destination image (ADI), and overall destination image (ODI). In this structure, CDI refers to the perception of potential tourists towards the attributes of tourist destinations (Echtner & Ritchie, 1993). ADI refers to the emotional response of potential tourists to various attributes of the destination, usually measured using a semantic differential scale (Russell et al., 1981). The cognitive destination image and ADI of tourists towards a destination together form the ODI, with the ODI being greater than the sum of its parts (H. Zhang et al., 2014). Moreover, these three components are theoretically ordered rather than independent: tourists first form a cognitive evaluation of a destination’s attributes (CDI), which subsequently elicits an affective response (ADI); that is, cognition precedes and shapes affect (Baloglu & McCleary, 1999; Beerli & Martin, 2004; Gartner, 1994). At present, the academic community agrees with the ‘new three-dimensional structure’ of DI and confirms that this structure is suitable for the Chinese cultural background (H. Zhang et al., 2011). Therefore, this article adopts the ‘new three-dimensional structure’ as the standard for dividing the measurement dimensions of the constituent elements of DI.

## 3. Hypothesis Establishment

### 3.1. The Effect of AIGP Scene Presentation

To clarify the link between scene presentation and CLT, we outline the theoretical reasoning underlying our hypotheses. According to CLT, panoramic-series AIGP convey a greater perceived spatial distance than close-up-series AIGP, and greater spatial distance is theoretically associated with a higher construal level, which emphasizes the central and desirable features of travel and may thereby foster a more positive destination image and stronger travel intention. We note that perceived spatial distance and construal level are treated here as theoretical constructs through which CLT interprets the expected effect; they were not directly measured in the present study. Accordingly, this reasoning is offered as a theoretical account of why panoramic-series AIGP may exert a stronger effect than close-up-series AIGP, rather than as an empirically tested causal chain. According to the theory of scene category subordination, this study divides the scene presentation of AIGP into close-up series and panoramic series. Specifically, the close-up series displays closer spatial distance and smaller visual range, with fewer visual elements included, while the panoramic series displays farther spatial distance and larger visual range, with more visual elements included (Artz et al., 1993). Based on CLT, this study suggests that close-up-series AIGP may make tourists perceive themselves as having a closer spatial distance to the scenery depicted in the AIGP and a lower level of abstraction of the scenery, leading to the use of a lower construal level to process information. Correspondingly, panoramic-series AIGP may make tourists feel that they are far away from the scenery in the AIGP, and the abstraction of the scenery is high, which may lead them to adopt a high construal level to process information. Since a high construal level emphasizes the central features and positive essence of an event (e.g., the overall value and emotional benefits of travel) while downplaying peripheral details and potential obstacles, it enhances individuals’ evaluation of future experiences and promotes behavioral intention (Trope & Liberman, 2010). In contrast, a low construal level focuses on concrete details and immediate concerns, which may inhibit intention formation by amplifying practical constraints. Thus, compared to close-up series AIGP, panoramic-series AIGP are likely to elicit more positive travel intentions. Meanwhile, overall information is inherently more powerful than local information (Miller, 1981). Just as significant and obvious stimuli exert more influence than weak and subtle ones, the abundant visual information in panoramic-series AIGP more effectively enhances tourists’ willingness and behavior. Thus, we assume the following:

**H1.**  *Compared to close-up-series AIGP, panoramic-series AIGP can evoke more positive travel intentions in tourists.*

Existing research indicates that information is the primary stimulus factor affecting the formation of DI (Baloglu & McCleary, 1999). In the study of spatial distance in advertising, it has been proven that the farther the information contained in the image is from the consumer, the more positive the consumer’s attitude becomes (Huang et al., 2017). Navon (1977) also believes that the perception of the global or overall theme is more realistic than the perception of individual elements. Therefore, panoramic-series AIGP are more conducive to inducing tourists to have a real perception of the destination, thereby shaping a more objective and positive impression. Thus, we assume the following:

**H2.**  *Compared to close-up-series AIGP, panoramic-series AIGP can elicit a more positive CDI in tourists.*

**H3.**  *Compared to close-up-series AIGP, panoramic-series AIGP can elicit a more positive ADI in tourists.*

### 3.2. The Chain-Mediating Effect of Destination Image

Research has shown that the characteristics of a product can affect the entire consumption process (Grinstein et al., 2019). Similarly, we believe that the scene presentation of AIGP can affect the entire process of tourists making subjective judgments and generating travel intentions. At present, the mediating effect of DI between external stimuli and tourist willingness has been widely explored and recognized (H. Zhang et al., 2014). Based on the ‘new three-dimensional structure’ of DI, the cognitive destination image and ADI of tourists towards the tourism destination jointly form the ODI, while CDI can also affect the ADI of tourists (Beerli & Martin, 2004). Building on the cognition–affect–overall ordering of destination image described in Section 2.3, we model CDI, ADI, and ODI as serial rather than as parallel mediators, such that scene presentation influences travel intention sequentially through CDI, ADI, and ODI, rather than through three independent parallel mediators. Meanwhile, the perception of DI has consistently been an important predictive variable influencing whether potential tourists will choose to travel to a destination (Phau et al., 2010). A positive ODI further enhances tourists’ willingness to travel. Building on our hypothesis that the scene presentation of AIGP directly affects DI and the underlying evidence mentioned above, this study assumes the following:

**H4.**  *The scene presentation of AIGP positively influences tourists’ travel intention through the mediation of CDI–ODI.*

**H5.**  *The scene presentation of AIGP positively influences tourists’ travel intention through the mediation of ADI–ODI.*

**H6.**  *The scene presentation of AIGP positively influences tourists’ travel intention through the mediation of CDI–ADI–ODI.*

### 3.3. The Moderating Effect of AIGP Source

How tourists perceive image content often depends on who provides the image. Research shows that, in UGC, differences in product type and presentation details typically do not have a significant impact on viewers’ perceptions (Shin et al., 2020). This is because people are more willing to trust UGC, and with this trust foundation, they no longer pay close attention to the details of the content, indicating that the impact of content source has a higher priority compared to content (Deng & Qu, 2022). In our research, based on CLT, tourists often have a greater psychological distance from the official perspective of OGC and a closer psychological distance to the personal sharing perspective of UGC. This is because UGC is perceived as a relatively independent and non-utilitarian third-party promotion, and it is more easily accessible to other users (Cheong & Morrison, 2008). Consequently, the primacy of source effect fundamentally alters the information processing mode. For UGC, the pre-established trust and psychological proximity create a heuristic processing framework in which nuanced cues from the content itself (e.g., the spatial distance implied by scene presentation) become less salient and are largely overlooked. The dominant ‘friend-sharing’ schema overshadows the subtler influence of visual composition. Therefore, the differential impact of close-up versus panoramic series on construal level—and subsequently on travel intention, CDI, and ADI—is attenuated. In contrast, for OGC, the lack of initial trust and greater psychological distance lead to more systematic and detailed processing of the content. In this context, the scene presentation (close-up vs. panoramic) becomes a critical cue for forming judgments, allowing its full effect on construal level and downstream variables to manifest.

**H7.**  *The AIGP source will moderate the effect of AIGP scene presentation on tourists’ travel intention, and this effect will be attenuated for UGC.*

**H8.**  *The AIGP source will moderate the effect of AIGP scene presentation on tourists’ CDI, and this effect will be attenuated for UGC.*

**H9.**  *The AIGP source will moderate the effect of AIGP scene presentation on tourists’ ADI, and this effect will be attenuated for UGC.*

**Figure 1:**
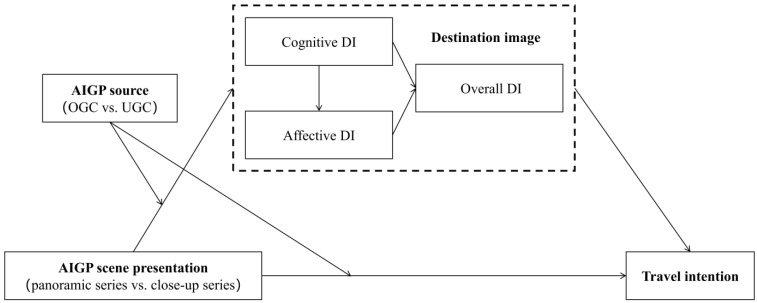
Research model.

We initiated this research with a pilot study, during which six sets of AIGP were evaluated to assess their impact on the proposed hypotheses. Based on the insights gathered, two sets of AIGP demonstrating the most significant effects were selected for further exploration in the main study. Study 1 investigated whether the scene presentation of AIGP influenced travel intention and explored the mediating role of DI. Study 2 replicated the findings of Study 1 and examined the potential moderating effect of AIGP source, comparing OGC and UGC. The current research framework is illustrated in Figure 1.

## 4. Research Methodology and Pilot Study

### 4.1. Scene Design

Chinese ancient towns are similar to hotels, exhibiting both hedonistic and utilitarian functions. When evaluating a hotel, people consider both utilitarian attributes related to safety and cleanliness, as well as hedonic attributes related to comfort and entertainment (Ren & Nickerson, 2019). Similarly, ‘ancient towns’ in China can be considered as tourist destinations that combine accommodation and leisure activities, where tourists gather for both utilitarian and entertainment purposes. Based on this, we choose ancient towns as the tourism scenes presented in the AIGP, which can eliminate consumer preferences for products with different decision-making methods. Meanwhile, a significant number of ancient towns have been developed for tourism in China, making them a popular choice for short-term residential travel across the entire country (Zou et al., 2023). This extensive development allows ancient towns to cater to the travel preferences of individuals from various regions, thereby mitigating the impact of destination accessibility on tourist preferences.

Midjourney is the AI image generation tool that we used in this study. Generally speaking, Midjourney closely approximates the appeal of real photographs, making it highly suitable for generating travel images (Göring et al., 2023). Furthermore, our research indicates that both individual and official users have previously utilized Midjourney to generate images on various social media platforms, making it an ideal fit for our research scenario. By using Midjourney’s prompt words to generate AIGP, we can preliminarily control variables such as pixel count and color temperature between different images, ensuring consistency for experimental purposes.

### 4.2. Data Collection

Prior to data collection, the required sample size was determined using G*Power 3.1 (F tests). For Study 1, which employed a one-way between-groups ANOVA comparing the panoramic and close-up series, an a priori analysis (ANOVA: fixed effects, omnibus, one-way) assuming a medium effect size (f = 0.25), with α = 0.05 and power = 0.80, indicated a required sample of 128 participants. For Study 2, which employed a 2 (scene presentation) × 2 (source) between-groups design, an a priori analysis (ANOVA: fixed effects, special, main effects and interactions; numerator df = 1, four groups) with the same parameters (f = 0.25, α = 0.05, power = 0.80) indicated a required sample of 128 participants for detecting the interaction effect. The sample sizes obtained in Study 1 (N = 200) and Study 2 (N = 352) both exceeded these requirements, ensuring adequate statistical power for the analyses. Since anyone can be a potential tourist, we chose to conduct our research online to expand the scope of the sample coverage and increase the sample size as much as possible. Participants for the study were recruited through www.wjx.com, a leading online data collection platform in China. In exchange for participation, individuals were compensated approximately 5 yuan. The platform distributed the survey link to its registered members via purposive sampling, following client requests. Recent studies in tourism research have validated the reliability and representativeness of samples obtained from www.wjx.com, confirming their suitability for academic studies (Liang et al., 2024). The sample demographics are detailed in Table 1.

### 4.3. Measures

In our experiments, we used seven-point scale questionnaires to measure the DI and travel intention of tourists, utilizing relatively well-validated scales. DI consists of three dimensions, CDI, ADI, and ODI, as referenced in the research by Qu et al. (2011) and Jalilvand et al. (2012). Travel intention measurements are based on the research by W. Zhang et al. (2022). The questionnaire items are provided in Table A1.

### 4.4. Pilot Study

Before the experiments began, we conducted a pilot test of the scenery presented in the experimental images to select AIGP that accurately represent close-up and panoramic series. For this purpose, Midjourney was used to generate six scenic images featuring an ancient town as the background. The prompt used to generate these panoramic images was ‘A Chinese ancient water town at sunset, traditional Jiangnan architecture with grey-tiled roofs, a stone arch bridge over a calm river, red lanterns, warm golden light, reflections on the water, wide-angle panoramic view, photorealistic, high detail.’ The close-up-series stimuli were not generated from separate prompts; instead, they were created in Adobe Photoshop by cropping the central arch bridge region of the corresponding panoramic image, so that the close-up series presented a partial view of the same scene. To ensure comparability, all stimuli were standardized to the same dimensions and resolution, and no other visual attributes (e.g., color temperature or brightness) were altered during editing.

The pilot study recruited 100 participants online, including 50 for the close-up series group and 50 for the panoramic series group. Participants were asked to carefully observe the AIGP, and then, based on the measurements developed by Wang et al. (2024) and K. Kim et al. (2019), participants were invited to judge the AIGP scene series they viewed using a seven-point scale (1 = close-up series, 7 = panoramic series). Based on the results from the pilot study, we selected two groups of AIGP, Ancient Town A (F = 12.80, *p* < 0.01) and Ancient Town B (F = 26.80, *p* < 0.001), as the main experimental materials. These groups of AIGP, both featuring arch bridges, exhibited the most significant differences in perception of the scene series.

## 5. Study 1

The purpose of Study 1 is to test the main effect of AIGP scene presentation on tourists’ travel intention and DI, as well as the mediating effect of DI. Specifically, this study aims to verify hypotheses H1 to H6.

### 5.1. Method

After excluding participants with self-contradictory or identical answers, a total of 200 subjects participated in this experiment, of which 85 were male. Participants were randomly and evenly assigned to either the close-up-series group or the panoramic-series group.

**Figure 2:**
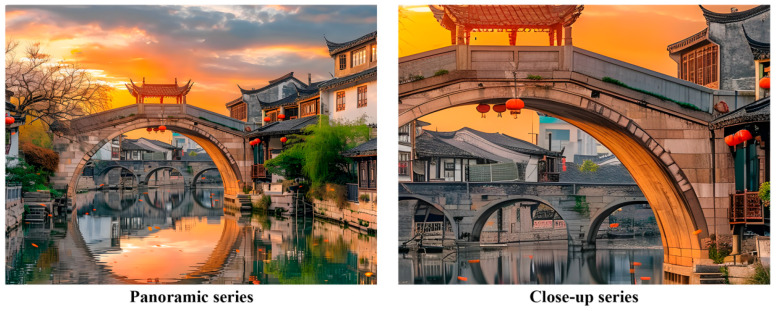
Image stimuli used in Study 1.

In the main study, we utilized two paired sets of AIGP that had previously been tested in the pilot study. These sets, featuring scenes from Ancient Town A, were specifically chosen to manipulate the scene presentation variable within the AIGP (see Figure 2). One set depicted all the scenes of an ancient town located by the river, with a bridge as the main feature (panoramic-series AIGP), while the other set included a part of the entire panoramic-series AIGP (close-up-series AIGP). In order to reduce the influence of brand power on tourists’ judgment, this virtual ancient town was named ‘Ancient Town A’. A fictional town was deliberately used so that participants held no prior brand associations or destination knowledge in order to isolate the effect of scene presentation. This is consistent with our definition of AIGP, which concerns the photorealistic visual style of the images rather than the real-world existence of the depicted scene. Additionally, when setting up the scenario, participants were informed through text that the price of, accessibility of, and travel time to such an ancient town were within their acceptable ranges. This was done to minimize the interference of individual tourist preferences on the experiment.

After being exposed to the stimuli, participants were instructed to complete a questionnaire measuring travel intention (α = 0.88) and DI, including CDI (α = 0.83) and ADI (α = 0.82). Additionally, we included the same manipulation check question from the pilot study, along with a range of demographic questions, in the questionnaire.

### 5.2. Results

Manipulation check: As determined by an ANOVA test, the two experimental groups showed significant differences in perception of the scene series (M_panoramic series_ = 4.90 vs. M_close-up series_ = 3.30; F(1, 198) = 40.36, *p* < 0.001, partial η^2^ = 0.169), revealing successful manipulation of the scene presentation in the AIGP.

Travel intention: One-way ANOVA revealed that the scene presentation of AIGP had a significant impact on travel intention. Specifically, participants assigned to the panoramic-series group demonstrated significantly stronger willingness to travel compared to those in the close-up-series group (M_panoramic series_ = 5.53 vs. M_close-up series_ = 4.99; F(1, 198) = 11.36, *p* < 0.01, partial η^2^ = 0.054).

Destination image: The results of a one-way ANOVA showed that, compared to those in the close-up-series group, participants in the panoramic-series group exhibited higher levels of CDI (M_panoramic series_ = 5.59 vs. M_close-up series_ = 5.13; F(1, 198) = 12.68, *p* < 0.001, partial η^2^ = 0.060), as well as higher levels of ADI (M_panoramic series_ = 5.61 vs. M_close-up series_ = 5.20; F(1, 198) = 9.43, *p* < 0.01, partial η^2^ = 0.045).

Chain-mediation effect analysis: Following (Hayes, 2017), model 6 in the bootstrap method was chosen for the mediation effect test. In the process, the sample size was set at 5000, and the confidence interval (CI) was set at 95%. The results indicated that the mediation effects of the three chain-mediation paths CDI–ODI (β = 0.1010, SE = 0.0420, 95%CI [0.0354, 0.1951]), ADI–ODI (β = 0.0775, SE = 0.0380, 95%CI [0.0171, 0.1634]), and CDI–ADI-ODI (β = 0.0339, SE = 0.0198, 95%CI [0.0060, 0.0811]) were all significant.

### 5.3. Discussion

The results from Study 1 showed that panoramic-series AIGP had a positive effect on participants’ travel intention and DI, supporting H1–H3. This is consistent with the study by Wang et al. (2024), which argued that panoramic views have a more positive effect on customer behavior than close-up views. Meanwhile, Study 1 also demonstrated the chain-mediated effect of DI, supporting H4–H6, suggesting an indirect association between AIGP scene presentation and travel intention through DI. Consistent with previous studies, we have demonstrated the interconnectedness of CDI, ADI, and ODI and the effect they jointly cause in the process of influencing tourists’ intentions (Qu et al., 2011).

## 6. Study 2

The purpose of Study 2 is to test the effect of different AIGP sources regulating the scene presentation of AIGP on tourists’ travel intention and DI, to validate H7–H9. At the same time, Study 2 is also expected to re-validate H1–H6 in order to increase the generalizability of the conclusions of Study 1 by replicating its results.

### 6.1. Method

A two-factor 2 (AIGP scene presentation: panoramic series vs. close-up series) × 2 (AIGP source: OGC vs. UGC) between-groups experimental design was used in Study 2. After excluding participants whose responses were self-contradictory or who provided identical answers throughout, 352 valid responses were retained, 143 of which were from male participants. Participants were randomly assigned to four experimental groups consisting of 88 participants each.

**Figure 3:**
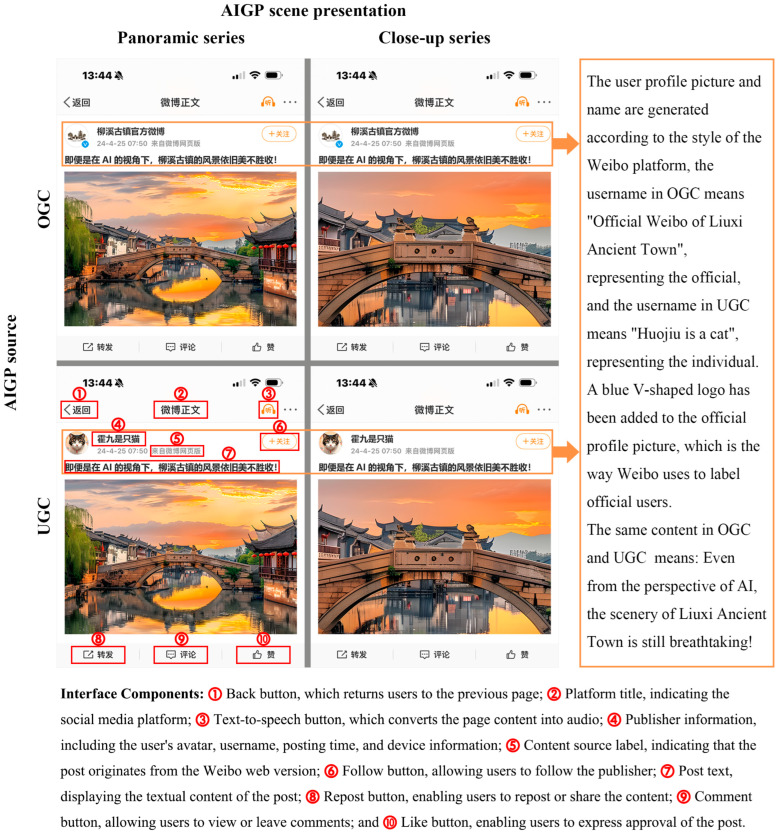
Image stimuli used in Study 2.

To manipulate the scene presentation of AIGP, two paired AI-generated ‘ancient town’ images previously tested in the pilot study (Ancient Town B) were employed, with the panoramic and partial views of the same image used as the panoramic and close-up series, respectively. To manipulate the AIGP source, both official and private account postings were created using Weibo, a major Chinese social media platform, as a backdrop. The OGC condition was presented as a post from an official account (the ‘Official Weibo of Liuxi Ancient Town’), marked with a blue ‘V’ verification badge in the way Weibo labels official accounts; the UGC condition was presented as a post from a personal account (‘Huojiu is a cat’), representing an individual user. The two conditions were identical in image content and caption, differing only in the account identity. In order to reduce the influence of brand power on tourists’ judgment without affecting their typical perceptions of social media, we followed the content style of the Weibo platform and named the AI-generated virtual town ‘Liuxi Ancient Town,’ which is a name that conforms to the naming convention of ancient towns in China. In addition, when setting up the scenarios, we designed them to fit the account style of the Weibo platform (see Figure 3) and informed the participants that the price of, accessibility of, and traveling time to the town were within their respective acceptance ranges through textual descriptions, so as to reduce the interference of personal preferences on the experimental results.

Following exposure to the stimuli, participants were asked to fill out a questionnaire measuring travel intention (α = 0.84) and DI, including CDI (α = 0.78) and ADI (α = 0.77). In addition, a number of demographic questions and the same manipulation check question from the pilot study were included in the questionnaire.

### 6.2. Results

Manipulation check: Participants in the panoramic-series and close-up-series groups showed significant differences in perceived scene series based on the ANOVA tests (M_panoramic series_ = 4.94 vs. M_close-up series_ = 3.49; F(1, 350) = 55.19, *p* < 0.001, partial η^2^ = 0.136), demonstrating the success of the scene presentation manipulation of AIGP.

**Figure 4:**
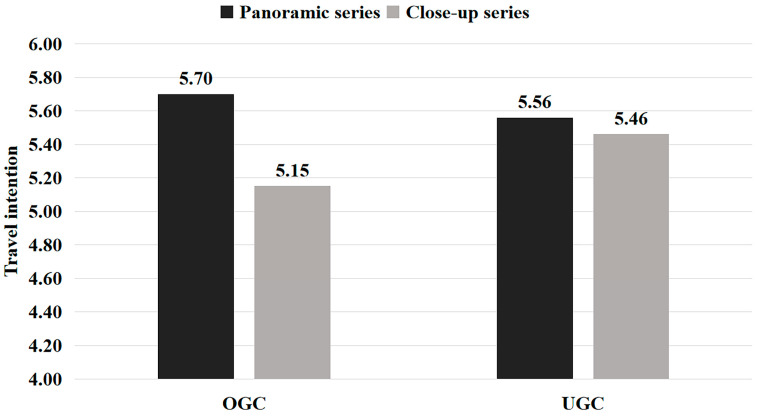
Comparison of travel intention means in Study 2.

Travel intention: Two-way ANOVA revealed a significant main effect of AIGP scene presentation (F(1, 348) = 7.86, *p* = 0.005, partial η^2^ = 0.022) and a significant interaction effect between AIGP scene presentation and AIGP source on travel intention (F(1, 348) = 5.16, *p* = 0.024, partial η^2^ = 0.015). Specifically, for the OGC, panoramic-series AIGP increased travel intention (M_panoramic series_ = 5.70 vs. M_close-up series_ = 5.15; F(1, 174) = 12.96, *p* < 0.001, partial η^2^ = 0.069). For the UGC, the effect of AIGP scene presentation on travel intention was mitigated (M_panoramic series_ = 5.56 vs. M_close-up series_ = 5.46; F(1, 174) = 0.15, *p* = 0.696, partial η^2^ = 0.001). See Figure 4.

**Figure 5:**
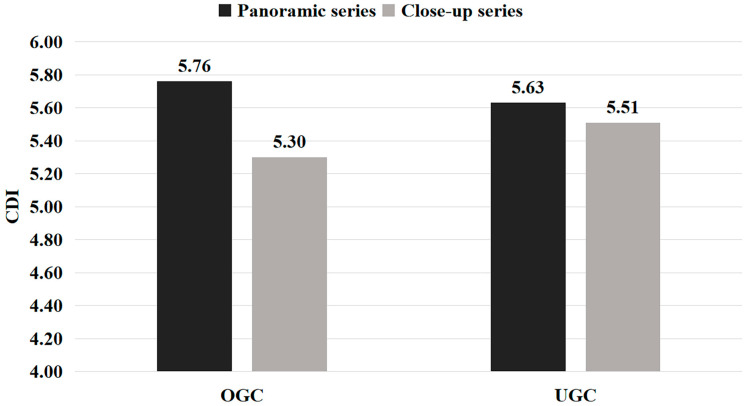
Comparison of CDI means in Study 2.

**Figure 6:**
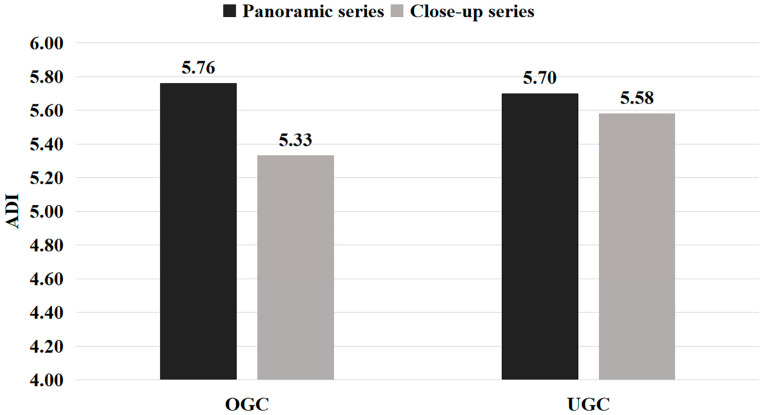
Comparison of ADI means in Study 2.

Destination image: Similarly, two-way ANOVA revealed a significant main effect of AIGP scene presentation (F(1, 348) = 11.40, *p* = 0.001, partial η^2^ = 0.032) and a significant interaction effect between AIGP scene presentation and AIGP source on CDI (F(1, 348) = 3.91, *p* = 0.049, partial η^2^ = 0.011). At the same time, two-way ANOVA revealed a significant main effect of AIGP scene presentation (F(1, 348) = 9.07, *p* = 0.003, partial η^2^ = 0.025) and a non-significant interaction effect between AIGP scene presentation and AIGP source on ADI (F(1, 348) = 2.98, *p* = 0.085 > 0.05, partial η^2^ = 0.008). Specifically, for the OGC, panoramic series AIGP increased CDI (M_panoramic series_ = 5.76 vs. M_close-up series_ = 5.30; F(1, 174) = 13.42, *p* < 0.001, partial η^2^ = 0.072) and ADI (M_panoramic series_ = 5.76 vs. M_close-up series_ = 5.33; F(1, 174) = 11.01, *p* < 0.01, partial η^2^ = 0.060). For the UGC, the effect of the AIGP scene presentation on CDI (M_panoramic series_ = 5.63 vs. M_close-up series_ = 5.51; F(1, 174) = 1.05, *p* = 0.307, partial η^2^ = 0.006) and ADI (M_panoramic series_ = 5.70 vs. M_close-up series_ = 5.58; F(1, 174) = 0.84, *p* = 0.360, partial η^2^ = 0.005) was non-significant. Figure 5 and Figure 6 present these results.

Chain-mediation effect analysis: Referring to (Hayes, 2017), model 6 in the bootstrap method was selected for the mediation effect test. For the operation, the sample size was set to 5000, and the confidence interval (CI) was set to 95%. In the OGC group, the results showed that all three chained mediation pathways were significant, including CDI–ODI (β = 0.0584, SE = 0.0215, 95% CI [0.0228, 0.1071]), ADI–ODI (β = 0.0535, SE = 0.0214, 95% CI [0.0163, 0.1015]), and CDI–ADI–ODI (β = 0.0153, SE = 0.0081, 95%CI [0.0035, 0.0355]). By contrast, in the UGC group, none of the mediation effects were significant, and the CIs all included 0. See Table 2 for the results.

### 6.3. Discussion

The results from Study 2 indicate that AIGP source moderated the effect of AIGP scene presentation on travel intention and CDI, but not on ADI. Specifically, the findings suggest that, for OGC, panoramic-series AIGP can contribute to a more positive CDI, leading to higher travel intention, and that this effect is attenuated for UGC (H7 and H8). This is in line with the study by H. Zhang et al. (2018), which suggests that officially published content has a more positive impact on viewers’ behavior compared to personally published content. However, the AIGP source did not show a significant moderating effect on the relationship between AIGP scene presentation and ADI; thus, H9 was not supported. This may be because the formation of people’s ADI is more influenced by their intuition towards the target image itself, whereas the image’s source identity has little impact. Consequently, the effect of AIGP scene presentation on ADI is not easily altered by the image’s source. Because the interaction effect on ADI did not reach significance (*p* = 0.085), we do not interpret it as a moderating effect. Taken together, our findings suggest that the AIGP source significantly moderated the effect of scene presentation on travel intention and CDI (supporting H7 and H8), but did not significantly moderate its effect on ADI (H9 not supported). Moreover, the direct effect of AIGP scene presentation in the OGC group in Study 2, as well as the chain-mediated effect of DI, further supported H1–H6.

## 7. General Conclusions and Discussion

Using two experimental studies, this research investigated the influence of scene presentation in AIGP. The results show that, compared to close-up-series AIGP, panoramic-series AIGP could increase tourists’ intention to travel to the destination. The mediation analyses further suggested that DI was statistically associated with the relationship between AIGP scene presentation and travel intention. Furthermore, the study delineated the moderating influence of the AIGP source, suggesting that the effect of AIGP scene presentation can be attenuated for UGC.

### 7.1. Theoretical Implications

Our research significantly contributes to the fields of tourism marketing and AIGC by confirming the impact of AIGP within the tourism context. It should be noted that we did not directly measure perceived psychological distance or construal level. CLT is therefore employed here as a theoretical lens to interpret the observed effects, rather than as an empirically tested mechanism. While existing research predominantly focuses on the effects of text produced by large language models such as ChatGPT, AI-generated images have garnered limited attention in this domain (Carvalho & Ivanov, 2024; Iskender, 2023). Furthermore, current studies of AI-generated images tend to concentrate on visual feature recognition, with scant exploration into how AI-generated images specifically affect viewers, such as tourists (Göring et al., 2023; Z. Zhang et al., 2023). Our study enriches the discourse on the role of visual communication in tourism and augments the AIGC literature by demonstrating that different forms of scene presentation in AIGP can indeed motivate tourists to visit a destination.

Second, this study contributes to the literature on visual cues by considering scene presentation in AIGP as a form of visual cue. Current studies of visual cues in pictures primarily focus on realistic photographs (Fink et al., 1996; H. Kim et al., 2024; F. S. Li & Ma, 2024), and only Su and Li (2024) have explored the unique role of spatial distance. Moving beyond real photography, we focused on the specific impact of scene presentation in AIGP, further expanding the unique insights into spatial distance within the realm of AI-generated images. By introducing DI as the psychological process through which AIGP scene presentation influences travel intention, our research extends prior research by exploring the impact of scene presentation as a visual cue in AIGP on tourists’ willingness to travel. We also provide empirical evidence on the effect of scene presentation on DI. Therefore, our study not only contributes to the literature on visual cues but also to the body of knowledge related to DI.

Finally, this study contributes to the literature on social media content sources. Currently, there is a wide range of research on content sources in social media, with some studies suggesting that UGC has a more positive and stronger influence on travel sharing than OGC, while others indicate that OGC has a stronger influence (Deng & Qu, 2022; H. Zhang et al., 2018). Additionally, there are findings that, in some cases, there is no discernible difference between the influences of the two (Shin et al., 2020). This paper is based on CLT and examines and finds that the influence of OGC is stronger in cases where the posted content is AIGP. We would like to clarify and point out that the way in which OGC and UGC influence viewers needs to be judged according to the scenario they are in. By exploring the moderating role of AIGP sources through the lens of CLT, this paper offers an additional perspective on OGC/UGC and social media content in destination marketing and extends the theoretical boundary of scene presentation research.

### 7.2. Practical Implications

Through meticulous empirical research, this study has several practical implications. Firstly, it provides empirical evidence for the specific effects of AIGP on DI and travel intention. Thus, the strategic use of AIGP by DMOs can foster positive impressions of destinations and enhance travel intentions. Specifically, based on the findings related to AIGP scenery, it is recommended that DMOs prioritize the use of panoramic-series AIGP in their promotional activities to more effectively attract tourists.

Secondly, this study has implications for the technological development of AI-generated images themselves. Notably, although some effects of scene presentation may be shared with conventional photographs, AIGP offer distinct practical value that ordinary photographs cannot: they can depict idealized, not-yet-built, conceptual, or fictional destinations and can preset angles, weather, lighting, and composition at low cost, overcoming the constraints of conventional photography. At the same time, AIGP carry generation-specific quality defects that are absent from real photographs. The findings suggest that visual cues in AIGP can influence tourists’ responses in ways that are consistent with prior findings from the conventional photograph literature, although direct comparisons between AIGP and traditional photographs remain necessary. According to CLT, the current limitations of AIGP are magnified when viewers focus more on details, potentially leading to adverse effects. Therefore, future developers of AI generation tools should consider that, when generating AI images, especially close-up series, greater attention should be paid to enhancing the image’s adequacy and detail. This improvement will help ensure that AI-generated images are better recognized and appreciated by viewers.

Finally, our research found that panoramic-series AIGP released by an official source is more effective, offering valuable insights for various social media platforms, including DMOs. For instance, if DMOs wish to use AIGP for promotion on social media, they can achieve better results by utilizing official channels to distribute panoramic-series AIGP. This approach leverages the broader and more impactful visual appeal of panoramic images to enhance viewer engagement and interest in the destination.

## 8. Limitations and Future Research

Our study also has some shortcomings and aspects that deserve further investigation. First, we investigated the specific effects of AIGP scene presentation on tourists’ perceptions and willingness, but comparisons between AI-generated images and conventional photographs still urgently need to be investigated. Future studies should directly compare AIGP with conventional photographs under identical scene presentations to isolate effects that are unique to AI-generated content, and the effects of other features of AIGP besides scene presentation could also be further explored.

Second, the close-up stimuli were created by cropping the panoramic images, because current AI image-generation tools cannot reliably produce two otherwise identical images differing only in scene presentation. Consequently, the panoramic and close-up series differed not only in spatial distance but also in visual information, aesthetic appeal, and composition, which co-vary with our intended manipulation. Moreover, we did not directly measure perceived psychological distance or construal level, and our manipulation checks confirmed only that participants distinguished the two series; construal level therefore cannot be empirically confirmed as the operative mechanism, and the observed effects may partly reflect these co-varying factors. In addition, the AI-generated nature of the stimuli may itself affect perceived psychological distance, a source-related dimension not separated from scene presentation in the present design. Future research should hold these visual dimensions constant, directly measure or manipulate construal level, and disentangle the AI origin of images from their scene presentation to more rigorously test the proposed mechanism.

Third, all mediators and the outcome variable were measured concurrently after participants viewed the stimuli. This cross-sectional measurement does not capture the temporal order among the variables, so the serial mediation paths should be interpreted as statistical associations consistent with theory rather than as established causal sequences. Longitudinal or multi-wave study designs that measure variables at different time points would allow stronger causal inferences.

Fourth, our experiments were conducted through online questionnaires, and both our samples and the ancient town tourism context were drawn entirely from China. Although we made efforts to preset the social media scenarios, this online self-report method may still introduce potential sample bias, and the Chinese samples and the particular appeal of ancient towns may limit the generalizability of the findings. Future research could employ field experiments or secondary data and extend the study to other populations and destination types beyond the Chinese context to control for destination characteristics similar to those of ancient towns.

## Figures and Tables

**Table 1:** Sample demographics.

Characteristics	Levels	Study 1 Percentage (%)	Study 2 Percentage (%)
Gender	Male	42.50	40.63
	Female	57.50	59.38
Age	<25	14.50	13.63
	26–30	56.00	56.53
	31–40	23.50	23.30
	>41	6.00	6.53
Education	High school and below	3.00	2.84
	Junior college	6.50	7.39
	Bachelor’s degree	80.00	81.53
	Master’s degree or above	10.50	8.24
Occupation	Student	9.50	7.67
	Public functionary	1.00	1.14
	Public institution staff	13.50	13.92
	Enterprise staff	70.50	71.02
	Self-employed	3.50	3.98
	Freelance	2.00	1.99
	Retiree	0.00	0.28
Monthly income	<¥3000	8.00	7.10
	¥3000–5000	16.50	15.34
	¥5000–10,000	41.50	45.17
	¥10,000–20,000	30.00	28.13
	>¥20,000	4.00	4.26

**Table 2:** Bootstrap results for the chain-mediation model in Study 2.

**OGC Group**	**Effect**	**Bootstrap Standard Error**	**95% CI**
CDI–ODI	0.0584	0.0215	[0.0228, 0.1071]
ADI–ODI	0.0535	0.0214	[0.0163, 0.1015]
CDI–ADI–ODI	0.0153	0.0081	[0.0035, 0.0355]
**UGC Group**	**Effect**	**Bootstrap Standard Error**	**95% CI**
CDI–ODI	0.0271	0.0179	[−0.0018, 0.0670]
ADI–ODI	0.0308	0.0210	[−0.0053, 0.0771]
CDI–ADI–ODI	0.0082	0.0069	[−0.0013, 0.0250]

## Data Availability

The raw data supporting the conclusions of this article will be made available by the authors on request.

## Note

1 https://mp.weixin.qq.com/s/1hfRyLqzF9e15ItTpvAwwg (accessed on 28 February 2024).

## Abbreviations

The following abbreviations are used in this manuscript:

AIGC	AI-generated content
AIGP	AI-generated photo
DMO	Destination marketing organization
CLT	Construal level theory
DI	Destination image
CDI	Cognitive destination image
ADI	Affective destination image
ODI	Overall destination image
UGC	User-generated content
OGC	Occupationally generated content
ANOVA	Analysis of variance

## Appendix A

**Table A1:** Constructs and measurement items.

Construct	Items	Source
Perceived scene presentation (1 = ‘close-up series’, 7 = ‘panoramic series’)	Do you think the image is a close-up-series shot or a panoramic-series shot?	Wang et al. (2024) and K. Kim et al. (2019)
Travel intention (1 = ‘strongly disagree’, 7 = ‘strongly agree’)	I think I will travel to the places that I viewed in the future.	W. Zhang et al. (2022)
	Compared to other travel destinations, I would prefer to visit the places that I viewed.	
	If I have the need to travel, I will visit the places that I viewed.	
	I will travel to the places that I viewed with my family or friends.	
Cognitive destination image (1 = ‘strongly disagree’, 7 = ‘strongly agree’)	The destination offers exciting and interesting places for visitors to explore.	Qu et al. (2011) and Jalilvand et al. (2012)
	The travel destination has a comfortable and pleasant environment.	
	The travel destination features beautiful scenery.	
	The travel destination has a rich tourism atmosphere.	
	As a travel goal, the destination is worth the price of admission.	
Affective destination image (1 = ‘strongly disagree’, 7 = ‘strongly agree’)	Traveling to this destination is comfortable and pleasant.	
	Traveling to this destination is uplifting.	
	Traveling to this destination is exciting.	
	Traveling to this destination is relaxing.	
Overall destination image (1 = ‘strongly disagree’, 7 = ‘strongly agree’)	I have a very good overall impression of the travel destination I viewed.	
